# Matrix Metalloproteinase-9 Is Involved in Chronic Lymphocytic Leukemia Cell Response to Fludarabine and Arsenic Trioxide

**DOI:** 10.1371/journal.pone.0099993

**Published:** 2014-06-23

**Authors:** Irene Amigo-Jiménez, Elvira Bailón, Estefanía Ugarte-Berzal, Noemí Aguilera-Montilla, José A. García-Marco, Angeles García-Pardo

**Affiliations:** 1 Cellular and Molecular Medicine Department, Centro de Investigaciones Biológicas, Consejo Superior de Investigaciones Científicas (CSIC), Madrid, Spain; 2 Hematology Unit, Hospital Universitario Puerta de Hierro, Madrid, Spain; Innsbruck Medical University, Austria

## Abstract

**Background:**

Matrix metalloproteinase-9 (MMP-9) contributes to chronic lymphocytic leukemia (CLL) pathology by regulating cell migration and preventing spontaneous apoptosis. It is not known if MMP-9 is involved in CLL cell response to chemotherapy and we address this in the present study, using arsenic trioxide (ATO) and fludarabine as examples of cytotoxic drugs.

**Methods:**

We used primary cells from the peripheral blood of CLL patients and MEC-1 cells stably transfected with an empty vector or a vector containing MMP-9. The effect of ATO and fludarabine was determined by flow cytometry and by the MTT assay. Expression of mRNA was measured by RT-PCR and qPCR. Secreted and cell-bound MMP-9 was analyzed by gelatin zymography and flow cytometry, respectively. Protein expression was analyzed by Western blotting and immunoprecipitation. Statistical analyses were performed using the two-tailed Student's t-test.

**Results:**

In response to ATO or fludarabine, CLL cells transcriptionally upregulated MMP-9, preceding the onset of apoptosis. Upregulated MMP-9 primarily localized to the membrane of early apoptotic cells and blocking apoptosis with Z-VAD prevented MMP-9 upregulation, thus linking MMP-9 to the apoptotic process. Culturing CLL cells on MMP-9 or stromal cells induced drug resistance, which was overcome by anti-MMP-9 antibodies. Accordingly, MMP-9-MEC-1 transfectants showed higher viability upon drug treatment than Mock-MEC-1 cells, and this effect was blocked by silencing MMP-9 with specific siRNAs. Following drug exposure, expression of anti-apoptotic proteins (Mcl-1, Bcl-xL, Bcl-2) and the Mcl-1/Bim, Mcl-1/Noxa, Bcl-2/Bax ratios were higher in MMP-9-cells than in Mock-cells. Similar results were obtained upon culturing primary CLL cells on MMP-9.

**Conclusions:**

Our study describes for the first time that MMP-9 induces drug resistance by modulating proteins of the Bcl-2 family and upregulating the corresponding anti-apoptotic/pro-apoptotic ratios. This is a novel role for MMP-9 contributing to CLL progression. Targeting MMP-9 in combined therapies may thus improve CLL response to treatment.

## Introduction

Chronic lymphocytic leukemia (CLL) is characterized by the accumulation of malignant CD5^+^ B lymphocytes in the peripheral blood and their progressive infiltration of lymphoid tissues [1], [2]. Frontline therapies for CLL consist in the administration of the purine analogue fludarabine, alone or in combination with other drugs such as anti-CD20 monoclonal antibodies or kinase inhibitors [3]–[5]. Because CLL is a heterogeneous disease, patients carrying specific molecular markers such as del17p13, unmutated IgV_H_ and/or high expression of ZAP-70 or CD38, do not respond well to these treatments [4], making it crucial to continue searching for new compounds useful in these cases. In this regard, arsenic trioxide (ATO), an efficient therapy in acute promyelocytic leukemia [6], [7], has been shown to induce apoptosis in all CLL cases including those with unfavorable prognosis [8]. We previously reported that the mechanism by which ATO induces CLL cell death is via c-jun N-terminal kinase activation and PI3K/Akt downregulation and this was observed in all samples tested, regardless of their prognostic markers [9]. ATO may thus constitute an efficient alternative/complementary treatment for CLL.

As with most tumors, CLL cell response to therapy is influenced by the microenvironment, whose cellular and molecular components provide survival signals that favor drug resistance [10], [11]. A consistent component of CLL niches is matrix metalloproteinase-9 (MMP-9) [12], which is also produced by CLL cells and upregulated by several stimuli [13]–[15]. Endogenous or/and exogenous MMP-9 binds to CLL cells via specific docking receptors and regulates cell migration [16]. Surface-bound MMP-9 also prevents CLL cell spontaneous apoptosis by a non-catalytic mechanism, consisting in Lyn/STAT3 activation and Mcl-1 upregulation [17], thus contributing to CLL progression.

It is not known if MMP-9 affects CLL cell response to chemotherapy. This is important to elucidate since MMP-9, as other MMPs, may play dual roles in apoptosis, either facilitating or antagonizing drug action [18], [19]. To approach this issue, we have studied whether MMP-9 is modulated by fludarabine or ATO treatment and whether it is involved in the CLL cell response to these compounds. Using primary CLL cells and a CLL-derived cell line stably expressing MMP-9 [20], we show that MMP-9 contributes to chemoresistance by preventing downregulation of anti-apoptotic proteins.

## Materials and Methods

### Patients, cells and cell culture

Approval was obtained from the CSIC Bioethics Review Board for these studies. All patients signed an informed consent before blood was drawn. B-lymphocytes were purified from the 20 CLL samples listed in Table 1 as reported [9], [17], using Ficoll-Paque PLUS (GE Healthcare, Uppsala, Sweden) centrifugation and, if necessary, negative selection with anti-CD3-conjugated Dynabeads (Invitrogen Dynal AS, Oslo, Norway). The resulting B cell population was mostly >90% CD19^+^, determined on a Coulter Epics XL flow cytometer (Beckman Coulter, Fullerton, CA). Primary stromal cells were obtained from a bone marrow sample of one CLL patient after 3 week culture in IMDM (Lonza, Amboise, France)/15% FBS, and used for up to 4 weeks. The HS-5 stromal cell line was obtained from Dr. Atanasio Pandiella (Cancer Research Center, Salamanca, Spain) and cultured in RPMI/10% FBS. The MEC-1 cell line, established from a CLL patient [21], was obtained from Dr. Enrique Ocio (Cancer Research Center, Salamanca), authenticated by short tandem repeat DNA typing (Secugen S.L., Madrid, Spain) and cultured in IMDM/10% FBS. MMP-9- and Mock-MEC-1 cells were generated by lentiviral transfection exactly as described [20]. Briefly, full-length human proMMP-9 DNA cloned in the pEGFP-N1 vector was amplified by PCR using cloned Pfu DNA polymerase (Agilent Technologies, Waldbronn, Germany) and inserted into the pCR-Blunt vector (Blunt Zero PCR cloning kit, Invitrogen). After restriction enzyme digestion, DNA sequences were inserted into the pRRL sin18.CMV.IRES.eGFP lentiviral vector (Dr. Juan Carlos Ramírez, Viral Vector Unit, Centro de Investigaciones Cardiovasculares, Madrid). Control constructs (Mock) contained only GFP DNA. Viral stocks were obtained after vector transfection of HEK293T cells and used to infect MEC-1 cells. GFP-expressing cells were selected by several cell sorting steps until more than 95% of the cells were clearly positive for expression. Cells were maintained in IMDM medium (Lonza, Basel, Switzerland), 10% fetal bovine serum (FBS).

**Table 1:** Clinical characteristics of CLL patients.

Patient	Sex/Age	Stage^a^	CD19^b^	CD5^b^	CD38/ZAP70^c^	Membrane MMP-9^b^	Ig Status	p53 status
**P1**	M/69	C/IV	88.0	11.2	−/+	15.8	ND	ND
**P2**	F/72	C/IV	95.0	97.6	−/+	18.4	Mutated	-
**P3**	M/70	B/II	91.5	99.5	+/+	2.5	Unmutated	-
**P4**	M/65	A/I	86.7	98.8	−/−	11.7	Mutated	-
**P5**	M/79	A/I	96.4	98.1	−/+	14.5	Unmutated	-
**P6**	M/79	B/II	96.2	83.2	+/−	6.6	Unmutated	-
**P7**	M/77	A/0	90.0	ND	+/ND	4.9	Unmutated	del+/mut+
**P8**	M/59	C/IV	95.9	86.6	+/+	1.0	ND	del+/mut+
**P9**	M/85	C/IV	91.5	5.0	+/ND	18.4	Unmutated	-
**P10**	F/73	A/II	97.3	26.2	−/−	1.9	Mutated	del-/mut+
**P11**	M/48	B/I	97.0	15.8	+/+	8.4	Unmutated	ND
**P12**	M/68	ND	95.6	26.6	+/ND	2.7	ND	-
**P13**	F/54	A/0	96.4	73.6	+/ND	15.8	Unmutated	-
**P14**	F/82	ND	95.3	52.9	+/ND	6.8	Unmutated	del-/mut+
**P15**	F/70	C/IV	93.0	91.0	+/ND	7.3	ND	ND
**P16**	F/ND	ND	93.1	95.1	+/ND	ND	ND	ND
**P17**	M/71	B/II	91.3	98.8	+/−	9.1	ND	-
**P18**	M/80	ND	90.1	44.1	+/ND	15.5	ND	ND
**P19**	M/62	ND	90.8	84.8	+/ND	11.5	ND	ND
**P20**	M/68	C/IV	90.7	83.7	−/ND	20.4	Mutated	-

a Clinical stage according to references [1], [2].

b Percentage of cells expressing this marker.

c The coexpression of CD38 and ZAP-70 has clinical prognostic value [1], [2].

ND, not determined.

### Antibodies and reagents

Rabbit polyclonal antibodies (RpAb) to MMP-9 (sc-6841R), Mcl-1 (sc-819), Bax (sc-526), Noxa (sc-52), Bcl-xL (sc-634), RhoGDI (sc-360), and mouse monoclonal Ab (mAb) to Bcl-2 (sc-509) were from Santa Cruz Biotechnology (Santa Cruz, CA). RpAb to Bim (559685) was from BD Pharmingen (Franklin Lakes, NJ). Rp IgG (isotype control for flow cytometry) was from Immunostep (Salamanca, Spain). mAb to vinculin (#V9131) was from Sigma-Aldrich (St. Louis, MO, USA). mAbs to CD19 and CD5 were from Diaclone (Besançon, France). mAbs against CD38 (16BDH), CD44 (HP2/9), α4 integrin subunit (HP2/1, function blocking), α4 integrin subunit (HP1/7, inactive control, isotype matched for HP2/1 and HP2/9), CD45, and β1 integrin subunit (Alex1/4) were from Dr. F. Sánchez-Madrid (Hospital de la Princesa, Madrid, Spain). HRP-labeled Abs to rabbit or mouse Ig (used for Western blotting) were from Dako (Glostrup, Denmark). Alexa 488- and Alexa 647-labeled Abs (used for flow cytometry) were from Molecular Probes (Eugene, OR). Rabbit TrueBlot (18-8816-31) was from Rockland Immunochemicals (Gilbertsville, PA). Bovine serum albumin (BSA) was from Roche Diagnostics GmbH (Mannheim, Germany). Propidium iodide (PI), MTT (3-(4,5-dimethylthiazolyl-2)-2,5-diphenyltetrazoliumbromide), actinomycin D, arsenic trioxide (ATO), fludarabine (2-fluoroadenine-9-β-D-arabinofuranoside) and the pan-caspase inhibitor Z-VAD-FMK were from Sigma-Aldrich. FITC-Annexin V was from Immunostep. MMP-9 was isolated from the conditioned medium of MMP-9-MEC-1 transfectants by gelatin-Sepharose affinity chromatography, as previously reported [17], [22]. Purity and identity of the protein was assessed by gelatin zymograhy and Western blotting analyses (see below).

### RT-PCR and RNA stabilization assays

Total RNA isolation and cDNA amplification were performed as described [17] using the following primers: MMP-9: 5′-TGGGCTACGTGACCTATGAC-3′ and 5′-CAAAGGTGAGAAGAGAGGGC-3′; c-*fos*: 5′-TACTACCACTCACCCGCAGA-3′ and 5′-CAGGTTGGCAATCTCGGTCT-3′; c-*jun*: 5′-CGACAAGTAAGAGTGCGGGA-3′ and 5′-CCCGTTGCTGGACTGGATTA-3′; glyceraldehyde-3-phosphate dehydrogenase (GAPDH): 5′- GGCTGAGAACGGGAAGCTTGTCA-3′ and 5′-CGGCCATCACGCCACAGTTTC-3′. PCR analyses were performed for 25 cycles consisting of 1 min denaturation-95°C, 1 min annealing-59°C, 1 min polymerization-72°C (MMP-9/GAPDH) or 30 s denaturation-95°C, 30 s anneling-60°C, 45 s polymerization-72°C (*c-fos/c-jun*). To assess mRNA stability, CLL cells were cultured with or without 3 µM ATO and after 20 h, 5 µM actinomycin D was added. At various time points samples were collected and mRNA levels of MMP-9 and GAPDH were measured by RT-PCR. Bands were visualized by ethidium bromide staining and quantified using the MultiGauge V3.0 program (Fujifilm Global Lifescience, Düsseldorf, Germany).

### Quantitative PCR (qPCR)

Quantitative PCR (qPCR) was performed using iQ SYBR Green Supermix (Bio-Rad Laboratories, Hercules, CA), and the primers described above for MMP-9. The primers for TATA binding protein (TBP) were: 5′-CGGCTGTTTAACTTCGCTTC-3′ and 5′-CACACGCCAAGAAACAGTGA-3′. Triplicate assays were performed, and results were normalized according to the expression levels of TBP RNA and expressed by using the ΔΔCT method for quantization.

### RNA interference experiments

The siRNA sequence targeting human proMMP-9 was: sense 5′-CAUCACCUAUUGGAUCCAAdTdT-3′ (targets bases 377–403); the siRNA sequence for negative control was: sense 5′-AUUGUAUGCGAUCGCAGACdTdT-3′. siRNA duplexes were verified to be specific for their targets by Blast search against the human genome and were custom-made by Sigma-Aldrich. 15×10^6^ Mock- or MMP-9-MEC-1 cells were nucleofected with 30 nM siRNAs using solution V and programme T-01 (Amaxa, Cologne, Germany), and assayed 24 h after transfection. Efficiency of transfection was confirmed by gelatin zymography and Western blot analyses.

### Cell viability assays

1.5×10^5^ CLL cells in RPMI/0.1% FBS were incubated with ATO, fludarabine or vehicle and cell viability was determined after 24 (ATO) or 48 (fludarabine) h on a Coulter Epics XL flow cytometer (Beckman Coulter, Fullerton, CA), using FITC-Annexin V and PI. In some experiments, live (Annexin V^−^/PI^−^) and early apoptotic (Annexin V^+^/PI^−^) cells were separated on a FACS Vantage cell sorter (Becton Dickinson, Franklin Lakes, NJ). For MTT assays, 0.75×10^5^ MEC-1 cells in IMDM/0.1% FBS were incubated with 50 µg MTT for 4 h in the dark. The blue MTT formazan precipitate was dissolved in isopropanol-HCl (24∶1) and the absorbance at 540 nm was determined on a Multiskan Bichromatic microplate reader (Labsystems, Helsinki, Finland).

### Cell fractionation

30×10^6^ cells CLL cells were serum-starved for 1 h and incubated with 3 µM ATO or vehicle for 24 h. Cells were collected, washed 1× with cold PBS, and incubated (15 min, 4°C) in 250 µl of ice-cold hypotonic digitonin buffer (5 mM Tris pH 7.5, 10 mM NaCl, 0.5 mM MgCl_2_, 1 mM EGTA, 20 µg/ml digitonin), containing a protease/phosphatase inhibitor cocktail (Roche Diagnostics). Cytosolic (soluble) and membrane (pellet) fractions were separated by centrifugation. The pellet was washed 1× with ice-cold PBS and extracted with 125 µl of NP-40 lysis buffer (10 mM Tris pH 7.5, 40 mM NaCl, 1 mM MgCl_2_, 1% NP-40, protease inhibitors). After 20 min at 4°C lysates were clarified by centrifugation. For MEC-1 transfectants, 5×10^6^ Mock- or MMP-9-cells were serum-starved for 3 h, washed with cold PBS, and incubated in 500 µl of ice-cold hypotonic digitonin buffer (containing 40 µg/ml digitonin). After separation of the cell fractions the pellet was extracted in 100 µl of NP-40 lysis buffer (containing 0.2% NP-40) and analysis continued as above. Soluble proteins in membrane and cytosolic fractions were quantitated using the Pierce BCA protein assay kit (Thermo Scientific, Rockford, IL) and analyzed by gelatin zymography. RhoGDI (typical cytosolic protein) and CD45 (typical membrane protein) were visualized by Western blotting and used as internal controls for the procedure.

### Gelatin zymography

The conditioned medium of ATO untreated or treated CLL cells was collected and concentrated 20× using ultrafiltration spin columns fitted with 30 kDa MWCO membranes (Sartorius Stedim Biotech GmbH, Goettingen, Germany). This medium, as well as membrane and cytosolic cellular fractions were analysed on 7.5% polyacrylamide gels containing 0.1% gelatin (Sigma-Aldrich). After electrophoresis gels were rinsed 3×30 min in 2.5% Triton X-100 and 1×30 min in distilled water, followed by overnight incubation in 50 mM Tris pH 7.5, 200 mM NaCl, 10 mM CaCl_2_ at 37°C. Gels were stained with 0.2% Coomassie blue and areas of gelatinolytic activity were visualized as transparent bands. Bands were quantitated using the MultiGauge V3.0 program.

### Analysis of MMP-9 protein expression

5×10^6^ CLL cells were cultured with or without 3 µM ATO (24 h) or 3 and/or 5 µM fludarabine (48 h). Secreted MMP-9 in the conditioned medium was analyzed by gelatin zymography. Surface-bound MMP-9 was determined by flow cytometry on the same cells, upon incubation with the anti-MMP-9 Ab or control rabbit IgG (1 h, 4°C), followed by Alexa 488- or 647- labeled Abs (20 min, 4°C). In some experiments, live (Annexin V^−^PI^−^) and early apoptotic (Annexin V^+^PI^−^) cells were separately analyzed for MMP-9 expression on a FACS Vantage cell sorter (Becton Dickinson, Franklin Lakes, NJ). Cells were also pre-incubated with anti-α4 integrin or anti-CD44 Abs for 1 h prior to ATO exposure, and subsequently analyzed for surface-bound MMP-9 by flow cytometry. Specific fluorescence (SF), also called “Generalized Integrated Mean Fluorescence Intensity (GiMFI) [23] and defined as mean fluorescence intensity (MFI)×% of positive cells, was chosen to represent MMP-9 expression. SF/GiMFI measurements have been previously used [24], as they may be more accurate than the individual MFI or % of positive cells values.

### Immunoprecipitation and Western blotting

For immunoprecipitation, 3×10^7^ Mock- or MMP-9-cells were serum-starved for 3 h and incubated with or without 5 µM ATO for 24 h. Cells were lysed in ice-cold 20 mM Tris-HCl pH 7.5, 150 mM NaCl, 1 mM EDTA, 0.5% NP-40, 1 mM PMSF, and protease inhibitors (IP buffer). After protein quantitation lysates were pre-cleared by incubating (1.5 h, 4°C) with 25 µl protein A-Sepharose (GE Healthcare Bio-Science, Uppsala, Sweden). Pre-cleared lysates were incubated (16 h, 4°C) with 3 µg primary or control Abs and mixed with 25 µl protein A-Sepharose for 2 h at 4°C. Pellets containing the immune complexes were washed with IP buffer and proteins extracted by boiling for 5 min in Laemmli buffer.

For Western blotting, samples were resolved by SDS-PAGE and transferred to nitrocellulose or PVDF membranes (Bio-Rad Laboratories, Hercules, CA). After electrophoresis, membranes were blocked with 5% BSA/TBS-Tween 20 for 1 h and incubated (4°C, 16 h) with primary Abs, followed by incubation for 1 h at room temperature with Rabbit TrueBlot HRP-labeled Abs (immune complexes) or HRP-labeled secondary Abs (whole lysates). To detect multiple proteins on the same membrane, after identification of the first protein, membranes were washed with TBS/0.1% Tween 20 for 10 min, followed by 3×30 min incubation in 1% glycine pH 2.2, 1% SDS, 0.0005% NP-40, at room temperature. Membranes were washed 1×10 min with TBS/Tween, blocked with 5% BSA for 1 h, and re-probed with subsequent primary and secondary Abs. Protein bands were developed using the enhanced chemiluminiscent detection method (Amersham) and quantitated as above.

### Statistical analyses

Normal distribution of the data was confirmed by the Shapiro-Wilk's normality test, using the univariate procedure of SAS 9.3 software (SAS Institute, Cary, NC). Statistical significance of the data was determined using the two-tailed Student's t-test. A p value of ≤0.05 was considered significant. Analyses were performed using the GraphPad InStat v3.06 software (GraphPad Software, San Diego, CA, USA). All values are expressed as means ± standard deviation, except for the qPCR assays in which means ± standard error are shown.

## Results

### ATO transcriptionally upregulates MMP-9 in CLL cells via c-fos/c-jun activation

**Figure 1:**
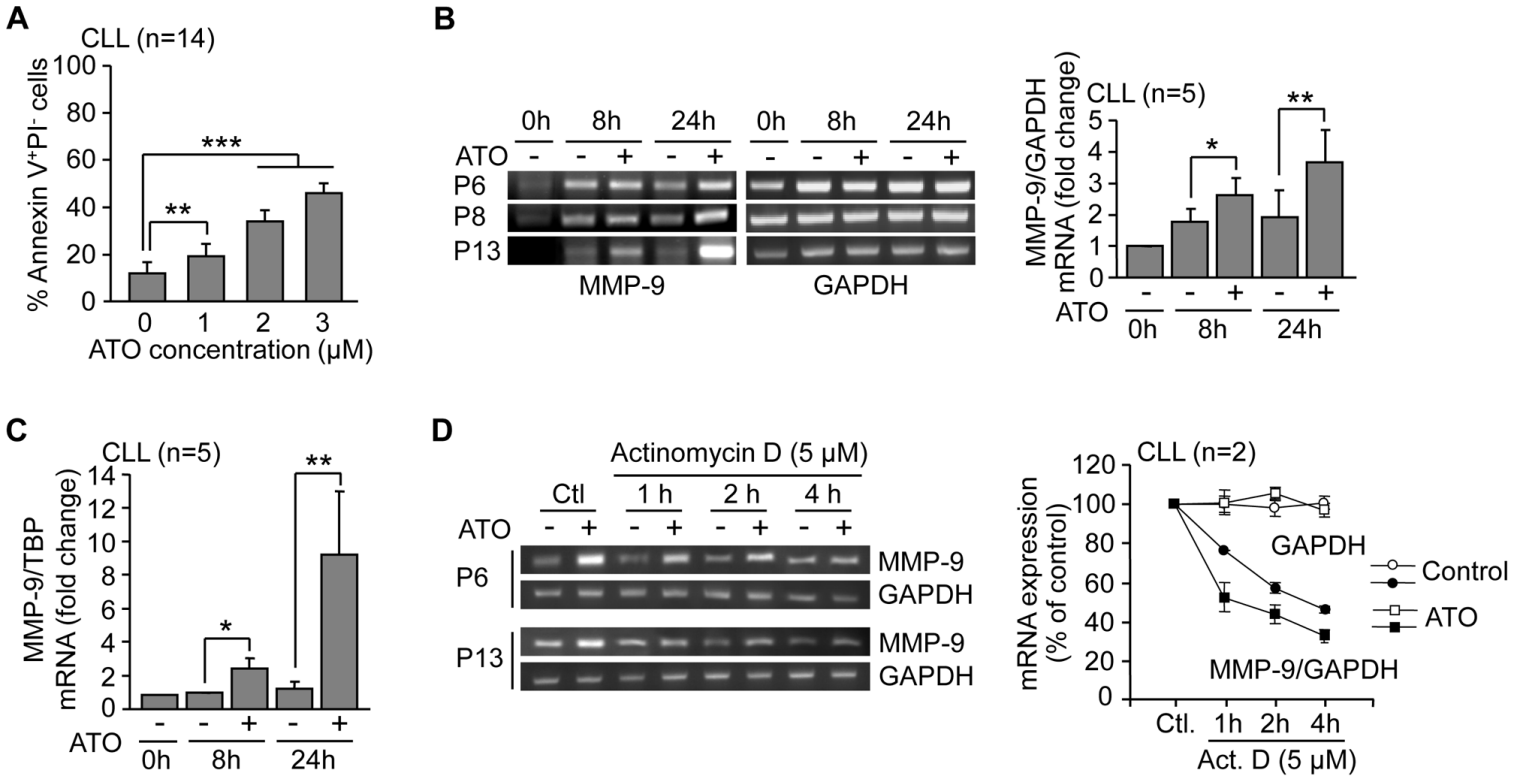
ATO transcriptionally upregulates MMP-9 in CLL cells. (A) 1.5×10^5^ CLL cells in RPMI/0.1%FBS were incubated with or without the indicated concentrations of ATO. After 24 h, cells were analyzed by flow cytometry using FITC-Annexin V and PI. (B) 10–15×10^6^ CLL cells were treated with 3 µM ATO for the indicated times and MMP-9 mRNA expression was analyzed by RT-PCR, using GAPDH mRNA as internal control. Normalized average values (fold change) are shown. (C) The same mRNA samples were also analyzed by qPCR using TBP as internal control and normalized average values (fold change) are shown. (D) 10–15×10^6^ CLL cells were cultured with or without 3 µM ATO for 20 h. Cells were then treated or not (Control, Ctl) with 5 µM actinomycin D and mRNA expression was analyzed at the indicated times. Values represent the average MMP-9/GAPDH ratio from the two samples after normalizing control values to 100. Values for GAPDH mRNA are also shown. *P≤0.05; **P≤0.01; ***P≤0.001.

To first establish the best conditions to study ATO action, CLL cells (1.5×10^6^/ml) were cultured for 24 h with or without various concentrations of ATO and apoptosis measured by flow cytometry, using FITC-Annexin V and PI. Figure 1A shows that the percentage of apoptotic cells (Annexin V^+^PI^−^) increased in a dose-dependent manner, reaching average values of 46.2% at 3 µM ATO (Figure 1A). 16.6% of the remaining cells were viable (Annexin V^−^PI^−^) and 37% were necrotic cells (Annexin V^+^PI^+^). As the 46.2% level of apoptosis seemed appropriate for biochemical studies, we chose 3 µM ATO concentration for subsequent experiments, except when indicated.

We next studied whether MMP-9, a protein previously shown to contribute to CLL survival [17], was modulated by ATO and involved in the cellular response to this agent. In initial experiments, 10–15×10^6^/ml CLL cells from three different patients were treated or not with 3 µM ATO and MMP-9 mRNA analyzed by RT-PCR. Figure 1B shows that MMP-9 mRNA expression increased after 8 or 24 h of ATO treatment, compared to control cells. Due to the endogenous MMP-9 production in CLL cell cultures, MMP-9 mRNA was also slightly elevated in untreated cells compared to constitutive values, with similar levels at 8 and 24 h (Figure 1B). The same samples were then analyzed by qPCR, which also showed a significant increase in MMP-9 mRNA expression of 2.4-fold and 9.3-fold, respectively, after 8 or 24 h treatment (Figure 1C). The average percentage of Annexin V^+^PI^−^ in ATO-treated cells at these times was 23.6% (8 h) and 37.6% (24 h), compared to 15% (8 and 24 h) in untreated cells (not shown).

To next study whether ATO affected the stability of MMP-9 mRNA, actinomycin D was added to CLL cells previously treated with 3 µM ATO for 20 h. RT-PCR analysis of these samples at various time points revealed that the increase in MMP-9 was not due to stabilization of MMP-9 mRNA (Figure 1D), indicating that ATO regulated MMP-9 at the transcriptional level.

**Figure 2:**
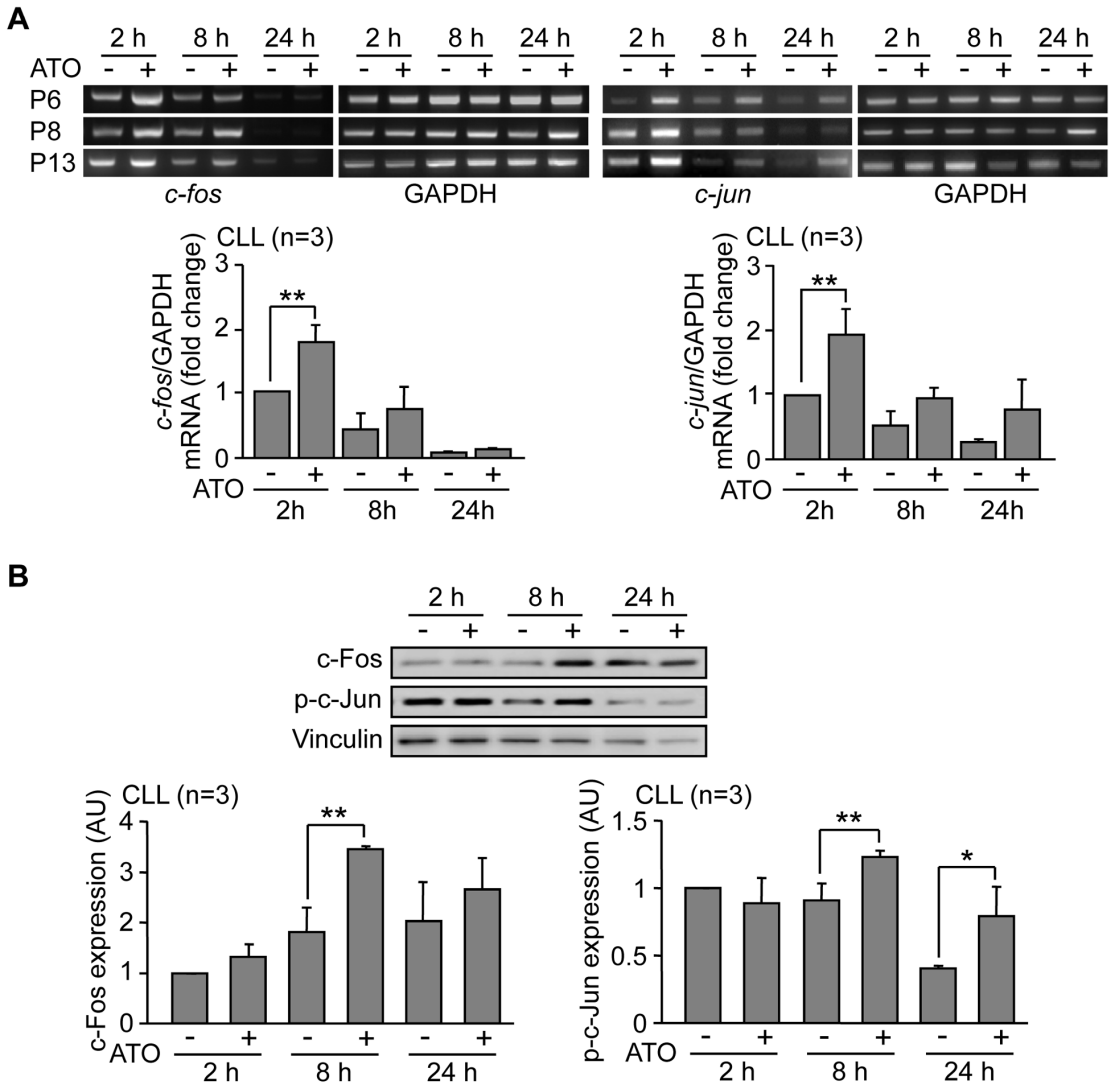
ATO treatment of CLL cells induces activation of the c-fos/c-jun transcription factors. (A) 10–15×10^6^ CLL cells in RPMI/0.1%FBS from three different patients were treated with 3 µM ATO or vehicle. At the indicated times, c-*fos* and c-*jun* mRNA was analyzed by RT-PCR, using GAPDH as an internal control. Average values (fold change) were normalized with respect to the control at 2 h. (B) 10–15×10^6^ CLL cells were treated as above, lysed at the indicated times and analyzed by Western blotting, using vinculin as internal control. The results for one representative sample and the normalized average values for the 3 samples studied, compared to the control at 2 h are shown. *P≤0.05; **P≤0.01.

Several transcription factors can activate the MMP-9 promoter including NF-κB and AP-1 [25]. Because we previously showed that ATO downregulates NF-κB and activates c-Jun N-terminal kinase (JNK) in CLL cells [9], we studied whether ATO regulated the JNK downstream effectors c-*fos* and c-*jun*, two components of the AP-1 heterodimer. RT-PCR analyses indicated that both, c-*fos* and c-*jun* mRNAs, were significantly upregulated upon cell exposure to 3 µM ATO, with maximun levels after 2 h (Figure 2A). At longer times expression declined but remained higher than control cells even at 24 h in the case of c-*jun* (Figure 2A). To determine whether this transcriptional modulation correlated with increased c-Fos and c-Jun protein expression, CLL cells from the same patients were treated with 3 µM ATO for various times, lysed and analyzed by Western blotting. Figure 2B shows the Western blot results for a representative sample and the average quantitation of the 3 patients studied. As observed, c-Fos increase was visible after 2 h, was maximal after 8 h and remained higher than the control after 24 h. Likewise, c-Jun phosphorylation was clearly observed after 8 h and remained elevated after 24 h of ATO treatment, compared to controls. As these times for c-Fos and phospho-c-Jun protein induction correlated with the upregulation of MMP-9 gene expression, these results strongly suggested that ATO regulated MMP-9 via AP-1 activation.

### ATO-induced MMP-9 mainly localizes at the cell membrane via interaction with α4β1 integrin and CD44

**Figure 3:**
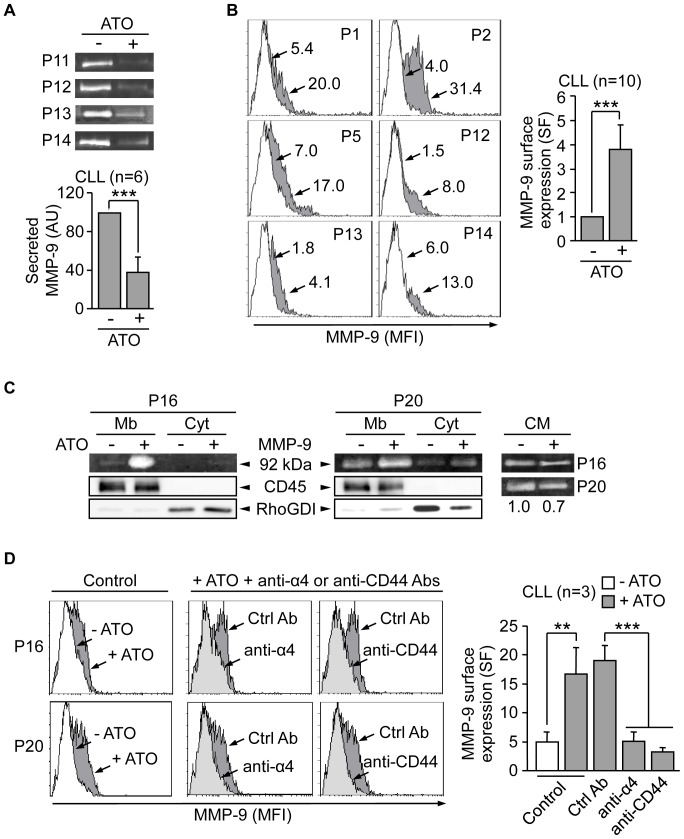
MMP-9 localizes to the CLL cell surface in response to ATO and in correlation with induction of apoptosis. (A) 5×10^6^ CLL cells in RPMI/0.1% FBS were treated or not with 3 µM ATO for 24 h. The conditioned media was collected, concentrated 20× and analyzed by gelatin zymography. The results from four representative samples and the average normalized values (arbitrary units, AU) from all six samples studied are shown. (B) 1.5×10^5^ CLL cells were incubated with or without 3 µM ATO for 24 h. MMP-9 surface expression was analyzed by flow cytometry using an anti-MMP-9 pAb or a control pAb. Histograms from six representative cases are shown, where white areas correspond to control/untreated cells and grey areas to ATO treated cells. Arrows indicate specific fluorescence (SF). Average normalized values from all ten samples analyzed are also shown. (C) 30×10^6^ CLL cells in RPMI/0.1%FBS were incubated with or without 3 µM ATO for 24 h. Membrane (Mb) and cytosolic (Cyt) fractions were separated and analyzed by gelatin zymography. RhoGDI and CD45 detected by Western blotting in the same lysates were used as controls for the procedure. The conditioned media (CM) of these cells was also analyzed by gelatin zymography and the normalized average values of the quantitated bands are shown (D) 1.5×10^5^ CLL cells in RPMI/0.1%FBS were incubated for 1 h with or without the indicated antibodies. 3 µM ATO was added and after 24 h, surface-bound MMP-9 was determined by flow cytometry using an anti-MMP-9 pAb or a control pAb. Histograms for two representative cases are shown. White areas correspond to untreated cells (−ATO) and grey areas (both light and dark) to ATO-treated cells (+ATO). Average normalized SF values are also shown. **P≤0.01; ***P≤0.001.

To determine if ATO also regulated MMP-9 at the protein level and because MMP-9 is mostly a secreted protein, we analyzed by gelatin zymography the conditioned media of equal number of CLL cells incubated with or without 3 µM ATO for 24 h. Figure 3A shows that, in contrast to what was expected, the levels of secreted MMP-9 in cells treated with ATO were significantly lower (2.6-fold average) than those in untreated cells. Since CLL cells also express MMP-9 on their surface [16] we studied whether cell-associated MMP-9 increased upon ATO treatment. CLL cells treated or not with ATO for 24 h were incubated with isotype control or anti-MMP-9 antibodies and analyzed by flow cytometry. The PI^+^ (necrotic) cell population was excluded from these analyses to avoid false results due to non-specific antibody capture. As shown in Figure 3B for 6 representative patients and quantitated for all 10 cases studied, surface-bound MMP-9 was significantly increased in cells treated with ATO (24% average positive cells) compared to control cells (10%). Moreover, cell fractionation analyses on two representative CLL samples confirmed that, upon ATO treatment, expression of MMP-9 was much higher in the membrane fraction than in the cytosolic fraction (Figure 3C). Parallel zymographic analyses of the conditioned media of the same samples confirmed that secreted MMP-9 was reduced on ATO-treated cells compared to controls (Figure 3C).

To then study whether the observed MMP-9 membrane association was via interaction with its reported receptors α4β1 integrin and CD44v [16], we blocked these receptors with specific antibodies prior to cell incubation with 3 µM ATO. As shown in Figure 3D for 2 representative patients and quantitated for the 3 cases studied, these antibodies significantly reduced the levels of MMP-9 found at the cell surface from 30.4% to 11.5% and 8.1%, respectively, for anti-α4 and anti-CD44 Abs upon ATO treatment. Moreover, these Abs also decreased cell viability by 19% (anti-α4) and 20% (anti-CD44) with respect to the effect of the control Ab (results not shown), suggesting a correlation between cell-bound MMP-9 and increased cell viability. In parallel gelatin zymography analyses of the conditioned media of these cells we did not observe an increase in soluble MMP-9, compared to control cells (not shown). This is likely due to the small variation of secreted MMP-9 under these conditions, with the consequent difficulty in quantitating differences.

**Figure 4:**
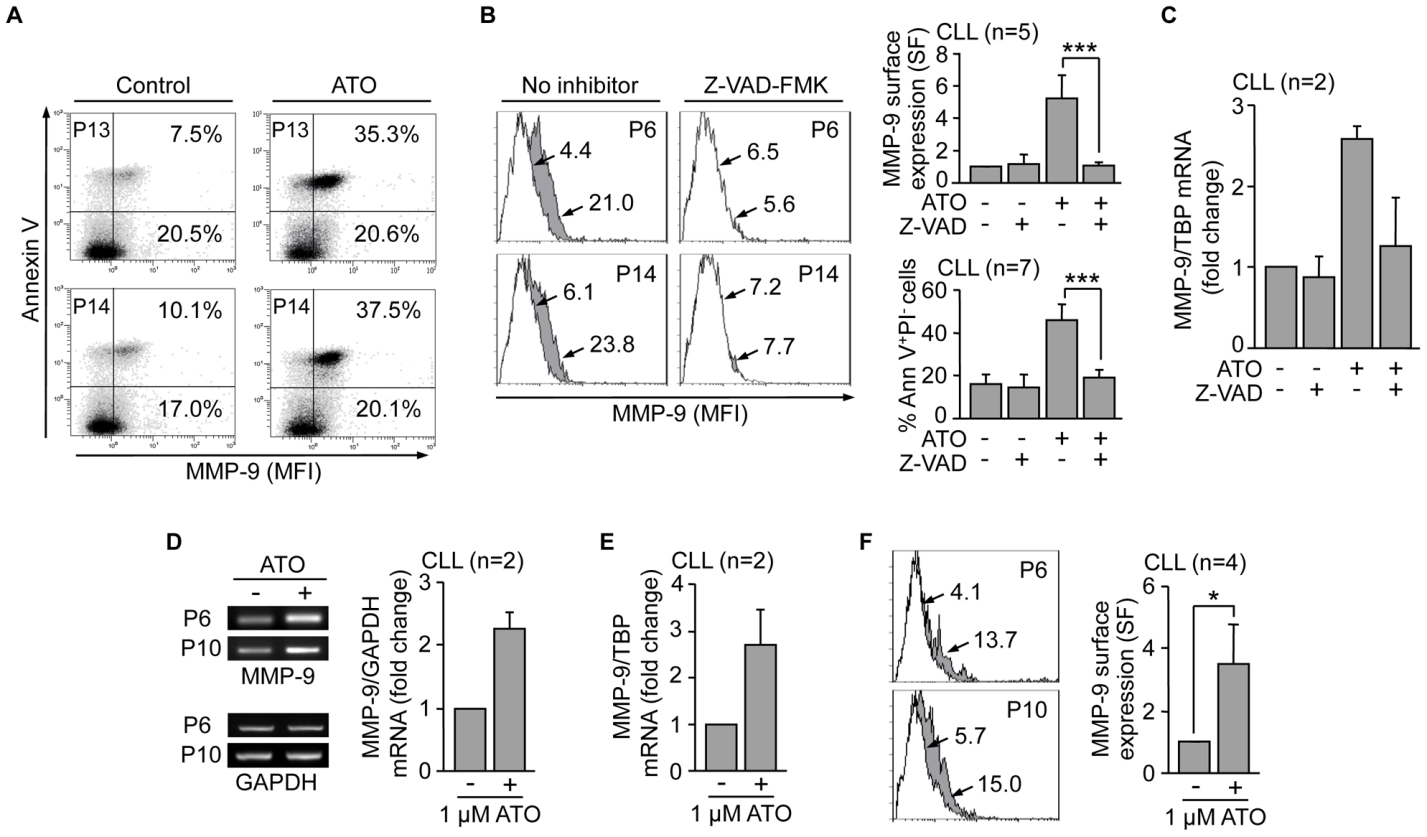
Upregulation and membrane localization of MMP-9 is an initial CLL cell response to the cytotoxic action of ATO. (A) Cell sorter biparametric diagrams of PI^−^ CLL cells (1.5×10^5^) treated or not with ATO for 24 h and analyzed for MMP-9 expression. Numbers indicate the percentage of cells expressing MMP-9 in the early apoptotic (Annexin V^+^, top) and live (Annexin V^−^, bottom) cell compartments. (B) Flow cytometric analysis of MMP-9 expression in control or ATO-treated CLL cells with or without previous incubation with 50 µM Z-VAD-FMK. Histograms from two representative cases are shown. White areas: control cells; grey areas: ATO-treated cells. Arrows indicate specific fluorescence (SF). Normalized average values for all five samples analyzed are shown. The average % of early apoptotic (Ann V^+^/PI^−^) cells in these samples is also shown. (C) 10–15×10^6^ CLL cells treated as in (B) were analyzed for MMP-9 mRNA expression by qPCR, using TBP as an internal control. Average normalized values (fold change) are shown. (D,E) 10–15×10^6^ CLL cells were treated with 1 µM ATO for 24 h and MMP-9 mRNA expression analyzed by RT-PCR (D) and qPCR (E). Normalized average values (fold change) are shown. (F) CLL cells treated as in (D, E) were analyzed for MMP-9 surface expression by flow cytometry with an anti-MMP-9 pAb or a control pAb. Histograms for the same samples used in (D, E) are shown. Arrows, white and grey areas are as in (B). Normalized average SF values of all four samples studied are shown. *P≤0.05; ***P≤0.001.

Next, we determined if increased transcription and surface expression of MMP-9 was associated to apoptosis, the main effect of ATO in CLL cells. To this end, live (Annexin V^−^PI^−^) and apoptotic (Annexin V^+^PI^−^) cells were analyzed on a cell sorter for MMP-9 surface expression after ATO exposure. The concentration of ATO in these experiments was adjusted to 2 µM to allow a more similar distribution between live and apoptotic cells and facilitate comparisons. Figure 4A shows for two representative cases that increased MMP-9 expression in response to ATO (35.3% and 37.5% positive cells, respectively, compared to 7.5% and 10.1%, respectively, in controls) was clearly coincident with the apoptotic cell population. To confirm these results CLL cells were incubated with the caspase-inhibitor Z-VAD-FMK prior to exposure to 2 µM ATO. This treatment prevented apoptosis and MMP-9 localization to the cell surface (Figure 4B). Moreover, the absence of membrane-bound MMP-9 correlated with the lack of MMP-9 mRNA upregulation by ATO, determined by qPCR analyses on these Z-VAD-FMK treated samples (Figure 4C).

To then determine if the observed MMP-9 gene induction and membrane localization preceded or was a consequence of the ongoing apoptosis, we lowered the ATO concentration to 1 µM, which had been previously shown to result in minimal apoptosis (see Figure 1A). Initial analysis by RT-PCR on 2 different samples clearly showed an increase in MMP-9 mRNA upon ATO treatment (Figure 4D). These results were further confirmed by qPCR, which showed a 2.4-fold average increase in MMP-9 mRNA for the 2 patients studied (Figure 4E). Moreover, flow cytometric analyses of these and 2 additional samples demonstrated the enhanced presence (from 8.8% to 20.4% positive cells) of MMP-9 at the cell surface (Figure 4F). Parallel viability analyses showed that, at the time studied, 1 µM ATO did not decrease cell viability with respect to control cells (P6: 75% vs 79%; P10: 54% vs 55%, not shown). Altogether these results suggested that CLL cells responded to an apoptotic stimulus like ATO, by first upregulating MMP-9 and its membrane localization. Upon the onset of apoptosis, MMP-9 remained specifically associated to apoptotic cells.

### MMP-9 upregulation and cell membrane localization in response to fludarabine treatment of CLL cells

**Figure 5:**
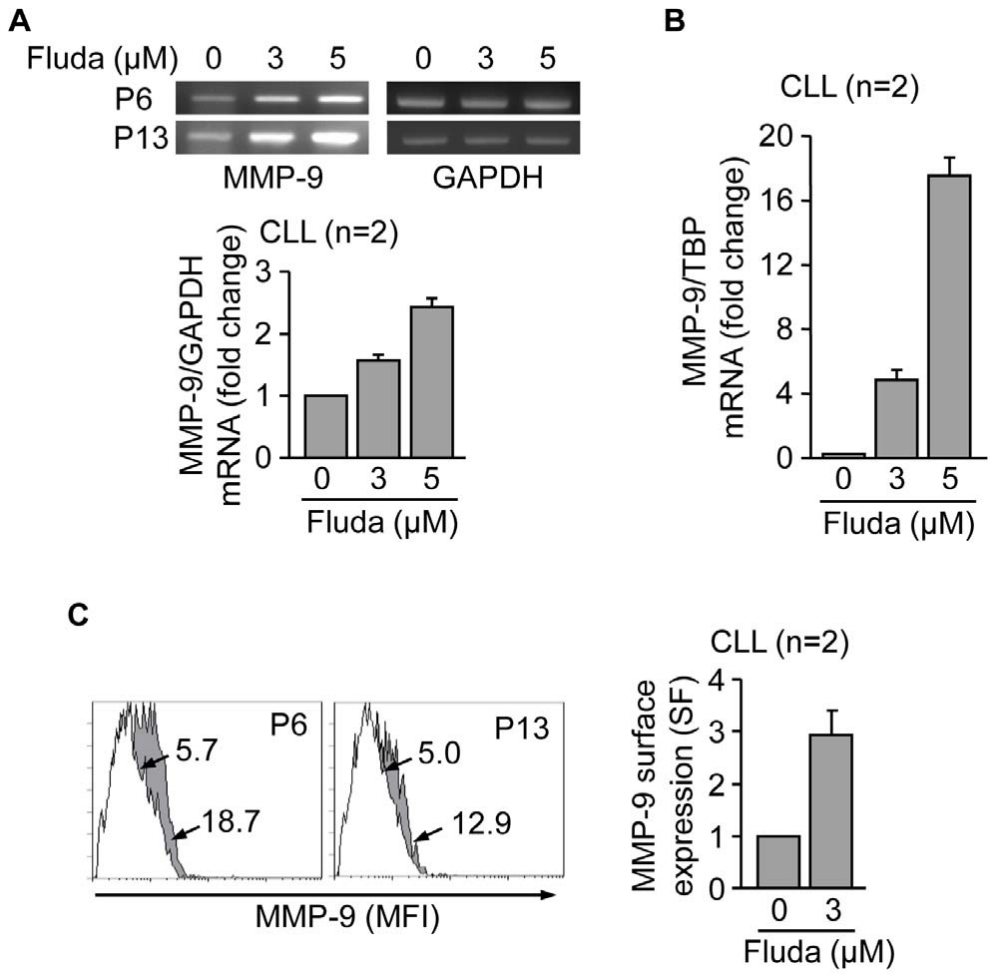
Fludarabine transcriptionally upregulates MMP-9 and induces its localization to the CLL cell membrane. (A,B) 10–15×10^6^ CLL in RPMI/0.1% FBS cells from two different patients were treated with 3 or 5 µM fludarabine (Fluda) for 48 h and MMP-9 mRNA expression was analyzed by RT-PCR (A) and qPCR (B). Normalized average values (fold change) are shown. (C) 1.5×10^5^ CLL cells from two different patients were incubated with or without 3 µM fludarabine for 48 h and MMP-9 surface expression was analyzed by flow cytometry. White areas, control/untreated cells; grey areas, fludarabine treated cells. Arrows indicate specific fluorescence (SF) values for each cell population. Normalized average values are also shown.

To determine whether MMP-9 modulation was a particular feature of ATO exposure or a more general response to drug-induced apoptosis, we studied the effect of fludarabine, a front-line treatment for CLL, on MMP-9. CLL cells were incubated with or without 3 or 5 µM fludarabine for 48 h and MMP-9 mRNA analyzed by RT-PCR. Figure 5A shows that fludarabine increased MMP-9 transcription in a dose-dependent manner, compared to control cells. These results were confirmed by qPCR, which showed an increase on MMP-9 mRNA of 4.9-fold and 17.5-fold, respectively, for 3 and 5 µM fludarabine (Figure 5B). Parallel flow cytometric analyses indicated that the average percentage of apoptotic cells at this time was 45.2% and 48%, respectively, for 3 and 5 µM fludarabine (not shown). As observed in the case of ATO, MMP-9 expression at the cell surface was enhanced (15.5% to 26.6% positive cells) upon fludarabine treatment (Figure 5C). These results indicated that MMP-9 upregulation in correlation with CLL cell apoptosis was not restricted to ATO action.

### MMP-9, isolated or present in stroma, induces resistance of CLL cells to ATO and fludarabine

**Figure 6:**
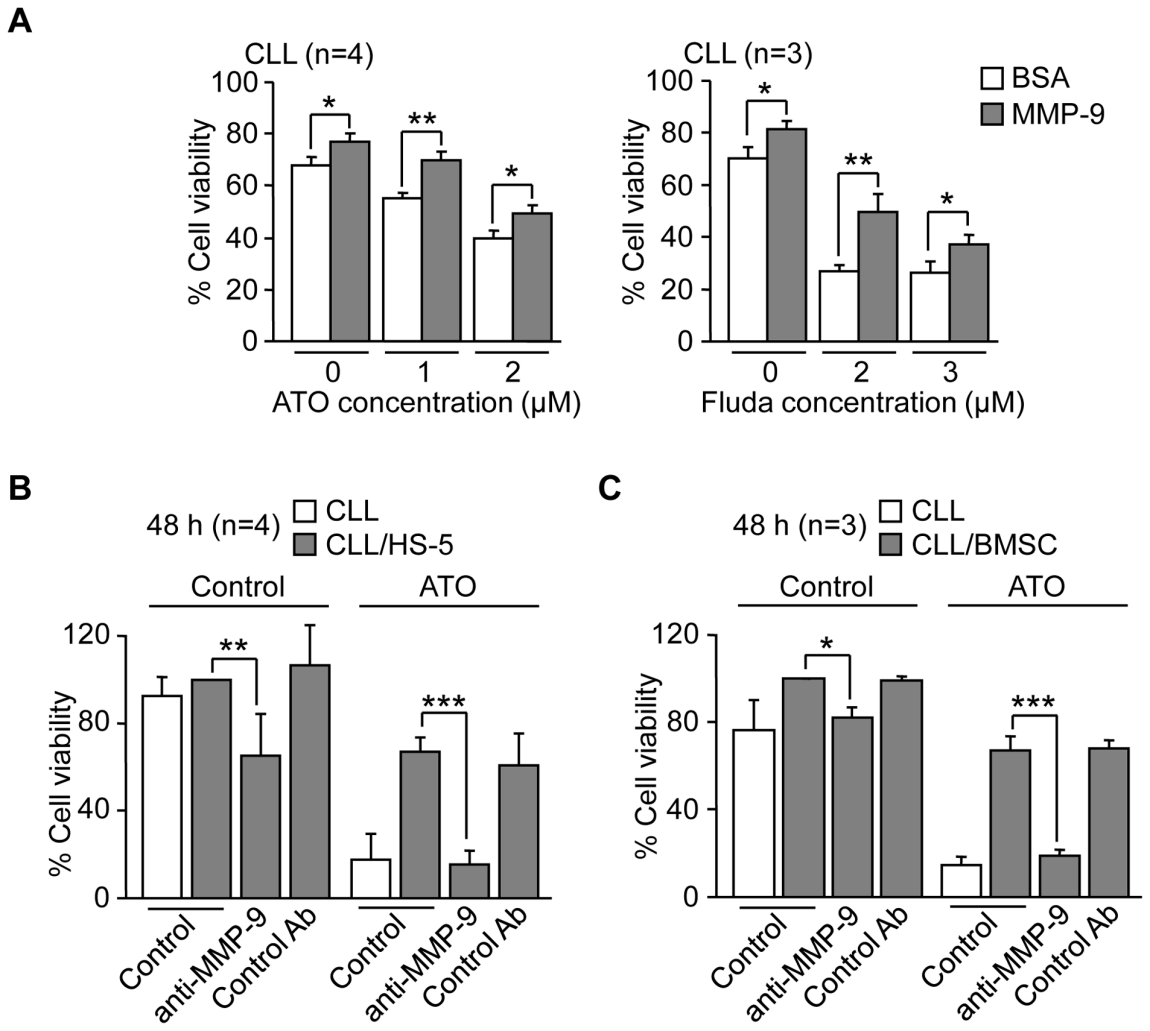
MMP-9, isolated or present in stroma, induces resistance of CLL cells to ATO and fludarabine. (A) 1.5×10^5^ CLL cells RPMI/0.1% FBS were cultured on 0.5% BSA or 150 nM MMP-9 for 1 h prior to adding the indicated concentrations of ATO or fludarabine (Fluda). After 24 h (ATO) or 48 h (Fluda) cell viability was determined by flow cytometry using FITC-Annexin V and PI. (B) 1.5×10^5^ CLL cells were treated or not with anti-MMP-9 pAbs or control pAbs for 1 h and added to wells coated with 0.5% BSA, HS-5 cells or primary stromal cells (BMSC). After 2 h at 37°C, 2 µM ATO was added and cells further incubated for 48 h. Cell viability was determined by flow cytometry using FITC-Annexin V and PI. The viability of CLL cells cultured over stroma in the absence of ATO was normalized to 100 and average values are shown. *P≤0.05; **P≤0.01; ***P≤0.001.

Having established that MMP-9 was modulated by ATO and fludarabine and localized to the CLL cell surface, we aimed to determine whether MMP-9 had a role in the cellular response to these drugs. This was particularly relevant, given the dual role played by MMPs in apoptosis [18], [19]. CLL cells were cultured on BSA (a control substrate that does not mediate cell adhesion or induce intracellular signaling) or MMP-9-coated wells for 1 h prior to exposure to ATO or fludarabine. Drug concentrations were lowered in these experiments to avoid excessive reduction in cell viability and allow comparisons. In control experiments in the absence of drug, MMP-9-cultures had significantly more live cells (Annexin V^−^PI^−^) than BSA-cultures (Figure 6A), in agreement with our previous results in which MMP-9 prevented CLL cell spontaneous apoptosis [17]. Importantly, cells cultured on MMP-9 and treated with ATO or, for comparison, with fludarabine, also showed significantly higher viability compared to cells cultured on BSA (Figure 6A).

The above results indicated that adhesion to MMP-9 induced CLL cell resistance to ATO and to the commonly used drug fludarabine. MMP-9 is an abundant component of the stroma found in the CLL microenvironment and stromal cells contribute to CLL cell resistance to certain drugs [10], [26]. We therefore studied whether stromal cells influenced the response of CLL cells to ATO and whether this involved MMP-9. CLL cells from 4 different patients were incubated on BSA (control) or HS-5 stromal cells and treated with or without ATO. Cell viability was determined after 48 h by flow cytometry and values for cells cultured on stromal cells in the absence of ATO (69.2% average) were normalized to 100. Figure 6B shows that in the absence of ATO, the anti-MMP-9 Ab significantly reduced the viability of CLL cells cultured on HS-5 cells compared to cells in the absence of Ab, while a control Ab had no effect. As in the case of isolated MMP-9 (Figure 6A), this confirmed our previous report showing that MMP-9 protects CLL cells from spontaneous apoptosis in culture [17]. Treatment with ATO reduced the viability of CLL cells cultured on BSA by 74.4% but had a limited effect (32.8% reduction) on HS-5 cultured-CLL cells (Figure 6B), indicating a protective effect by stromal cells. This was completely overcome by the anti-MMP-9 Ab, which reduced CLL viability to 15.5%, while a control Ab had no effect (Figure 6B).

To confirm and validate these results, the same experiments were carried out on CLL cells cultured on primary stromal cells derived from a CLL patient. Primary stromal cells protected CLL cells from spontaneous apoptosis (undergone in suspended cells) and this was significantly reverted by an anti-MMP-9 Ab, but not by a control Ab. Primary stromal cells also significantly induced CLL cell resistance to ATO (67.1% cell viability compared to 14.5% on suspended cells) and the anti-MMP-9 Ab clearly overcame this protective effect, reducing the stroma-induced survival to 18.8% (Figure 6C). Altogether these results established that stromal cells protected CLL cells from the cytotoxic effect of ATO and that MMP-9 had a role in this protection.

### MEC-1 cells also upregulate MMP-9 in response to ATO and fludarabine

**Figure 7:**
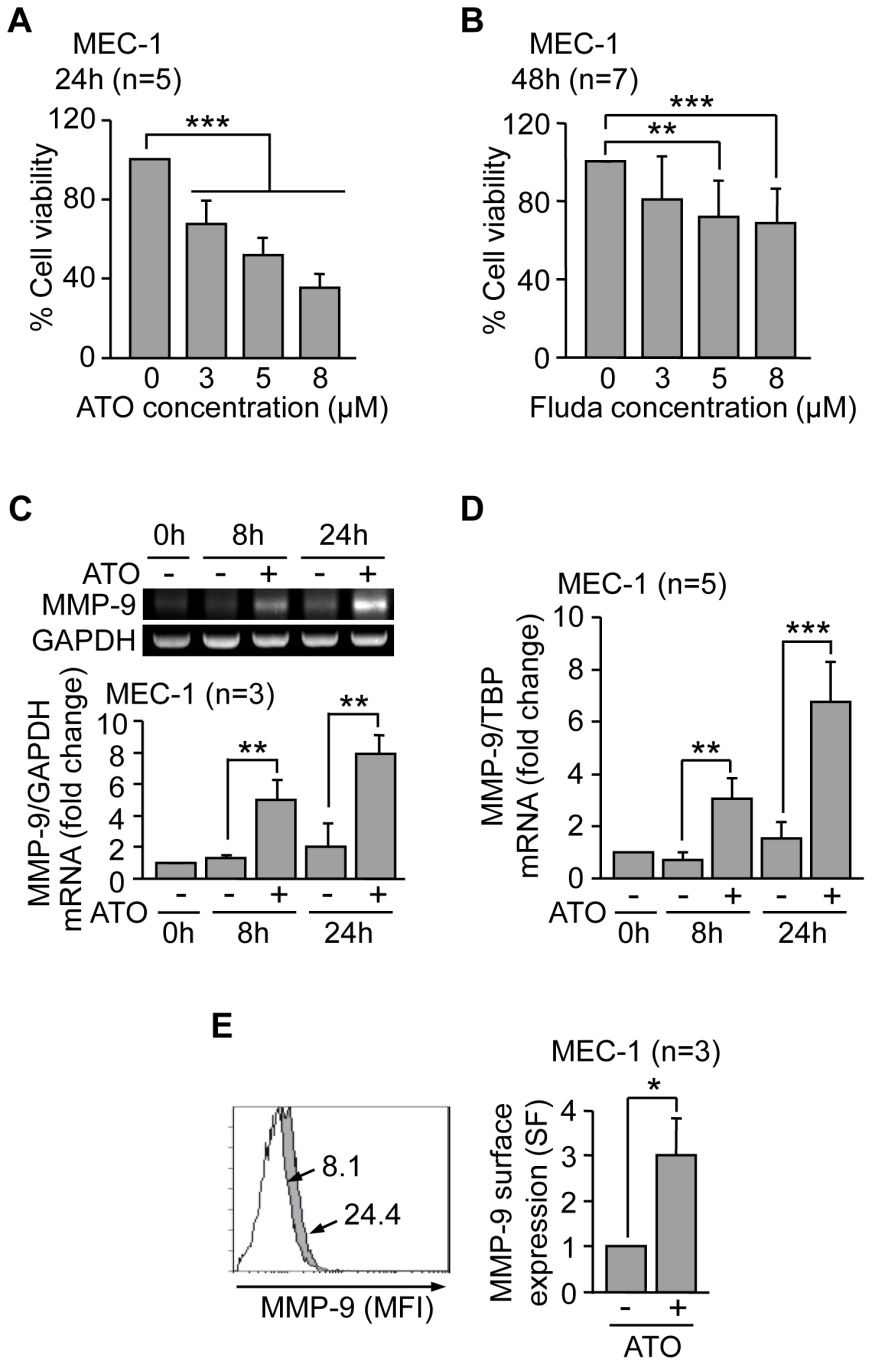
Effect of ATO and fludarabine on MEC-1 cells. (A, B) 7.5×10^4^ MEC-1 cells in IMDM/0.1% FBS were treated or not with the indicated concentrations of ATO (A) or fludarabine (Fluda) (B). After 24 h (ATO) or 48 h (Fluda), cell viability was determined by the MTT assay. Control cell viability was normalized to 100 and average values are shown. (C,D) 5×10^6^ MEC-1 cells were treated with 3 µM ATO for the indicated times and MMP-9 mRNA expression was analyzed by RT-PCR, using GAPDH as internal control (C) and qPCR, using TBP as internal control (D). Normalized average values (fold change) are shown. (E) 1.5×10^5^ MEC-1 cells were treated or not with 3 µM ATO for 24 h and MMP-9 surface expression was analyzed by flow cytometry, using an anti-MMP-9 pAb or a control pAb. Histograms from a representative experiment and normalized average values for the three experiments performed are shown. White areas, control/untreated cells; grey areas, ATO-treated cells. Arrows indicate the specific fluorescence. *P≤0.05; **P≤0.01; ***P≤0.001.

To further establish that MMP-9 conferred drug resistance in CLL cells we used the MEC-1 cell line, derived from a CLL patient and expressing very low constitutive levels of MMP-9. To first determine if these cells behave like primary CLL cells, we studied the response of MEC-1 cells to ATO and, for comparison, to fludarabine. The viability of untreated cells after 24 h and 48 h was 146% and 154%, respectively, compared to initial viability normalized to 100 (due to cell proliferation), and these values were normalized to 100. Figure 7A,B shows that after 24 h (ATO) or 48 h (fludarabine) treatment, the viability of MEC-1 cells, measured by the MTT assay, decreased in a dose-dependent manner. Because this assay primarily determines cell proliferation and, indirectly, cell viability, we also measured MEC-1 cell viability after ATO or fludarabine treatment by flow cytometry, using FITC-Annexin V and PI. In results not shown, ATO decreased cell viability by 39%, 58% and 79%, at 3, 5, and 8 µM, respectively. Likewise, fludarabine treatment reduced viability by 13%, 29%, and 32%, at 3, 5 and 8 µM, respectively. These results were very similar to those shown in Figure 7A,B, thus confirming the validity of the MTT assay to assess cell viability.

We next studied whether ATO also modulated MMP-9 in MEC-1 cells. Indeed, and as observed in primary CLL cells, ATO upregulated MMP-9 mRNA on MEC-1 cells after 8 and 24 h, compared to control cells (Figure 7C). These results were confirmed by qPCR, which showed a 4.2-fold and 4.4-fold MMP-9 mRNA increase, respectively, after 8 or 24 h treatment (Figure 7D). Moreover, MMP-9 surface expression on these cells also significantly increased (from 15.6% to 34.1% positive cells) upon ATO treatment (Figure 7E). These results confirmed the similar response of MEC-1 and primary CLL cells to ATO and validated the MEC-1 cell system for subsequent studies.

### MMP-9 expression in MEC-1 cells confers resistance to ATO and fludarabine

**Figure 8:**
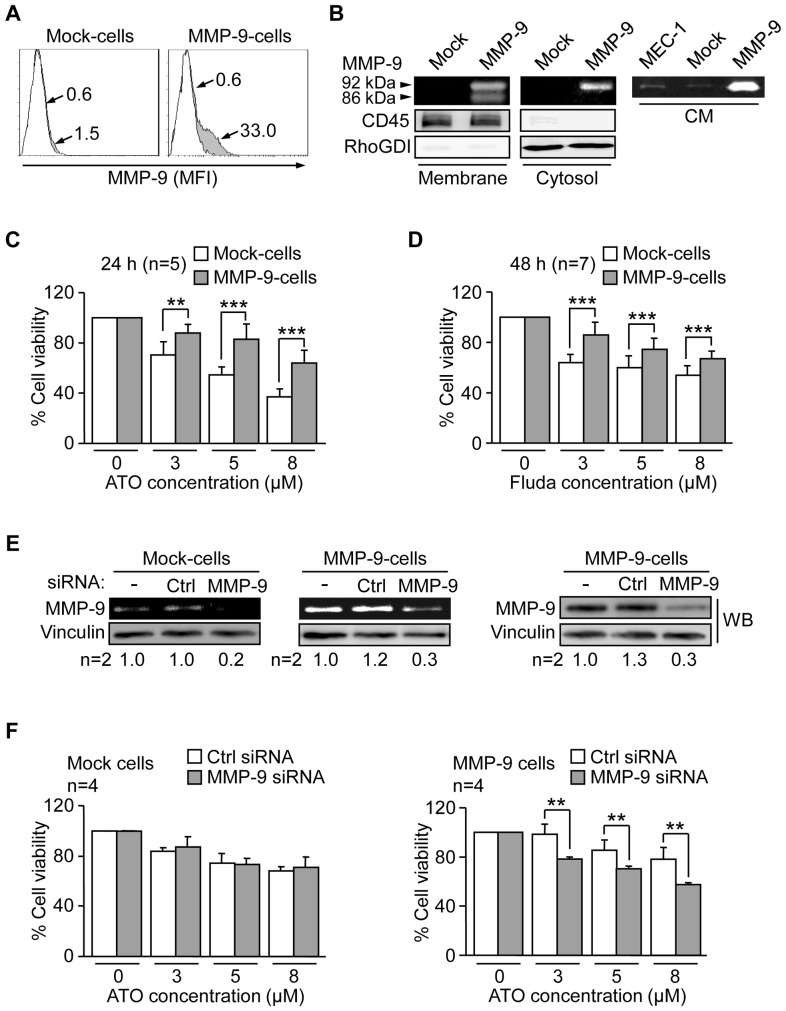
Effect of ATO and fludarabine on MEC-1 Mock-cells and MMP-9-cells. (A) 1.5×10^5^ Mock- or MMP-9-cells were analysed by flow cytometry using an anti-MMP-9 pAb or a control pAb. Histograms for two representative experiments are shown. White areas: negative control; grey areas: MMP-9 surface expression. Arrows indicate the specific fluorescence (SF). (B) The membrane and cytosolic fractions from 5×10^6^ Mock- or MMP-9-cells were separated and analyzed by gelatin zymography. RhoGDI and CD45 were visualized by Western blotting of the same lysates and used as controls for the procedure. The conditioned medium (CM) of the same cells was also analyzed by gelatin zymography. (C,D) 7.5×10^4^ Mock- or MMP-9- cells in IMDM/0.1% FBS were treated or not with the indicated concentrations of ATO (C) or Fluda (D) and cell viability was determined after 24 h (ATO) or 48 h (Fluda) using the MTT assay. The viability in the absence of drugs was normalized to 100 and average values are shown. (E) 15×10^6^ MEC-1 transfectants were nucleofected with MMP-9 or control siRNAs and analyzed after 24 h by gelatin zymography and Western blotting. Average quantitation of the MMP-9/vinculin ratios is also shown. (F) 7.5×10^4^ siRNA-transfected Mock- or MMP-9-cells were treated or not with the indicated concentrations of ATO. After 24 h cell viability was determined by MTT. The viability in the absence of drugs was normalized to 100 and average values are shown. **P≤0.01, ***P≤0.001.

Using the MEC-1 cell line, we recently established [20] stable transfectants expressing a GFP-lentiviral vector (Mock-cells) or a vector containing GFP-MMP-9 (MMP-9-cells), thus representing an unambiguous system to study MMP-9 functions. As recently reported [20], MMP-9-cells expressed cell-associated MMP-9, determined by flow cytometry (Figure 8A), thus resembling primary CLL cells [16]. This expression was further confirmed by cell fractionation, which clearly showed the presence of MMP-9 on the membrane (92 kDa and 86 kDa forms) and cytosol (92 kDa form) fractions of MMP-9-cells and its absence on Mock-cells (Figure 8B). Additionally, MMP-9-cells secreted high levels of MMP-9 into the medium, while secretion was very low in untransfected or MEC-1 Mock-cells (Figure 8B).

Mock- and MMP-9-cells where then incubated with various concentrations of ATO (24 h) or fludarabine (48 h) or vehicle and viability measured by the MTT assay. In the absence of drug, the viability of Mock-cells and MMP-9-cells was similar (155% vs 149% at 24 h and 154% vs 152% at 48 h, compared to initial viability) and these values were normalized to 100, for better assessment of the effect of the drugs. Treatment with ATO (Figure 8C) or fludarabine (Figure 8D) decreased cell viability in a dose-dependent manner in both cell types, but at all doses tested MMP-9-cells showed significantly higher viability than Mock-cells. To further confirm that this was due to the presence of MMP-9 (cell-associated and in soluble form), we performed gene silencing experiments using a specific siRNA for MMP-9 or a control siRNA. Gelatin zymographic analyses indicated that MMP-9 silencing inhibited the already low MMP-9 expression in Mock-cells, and produced an average 75% reduction in MMP-9-cells, also confirmed by Western blotting (Figure 8E). This procedure equally affected the viability of either transfectant cell type (35% reduction). siRNA-transfected Mock- and MMP-9-cells were then treated with several concentrations of ATO and the viability measured after 24 h by MTT. Although the effect of ATO was milder in these experiments, there were clear differences between Mock- and MMP-9-cells. As shown in Figure 8F, the viability of Mock-cells upon ATO exposure decreased in a dose-dependent manner with no differences between MMP-9 siRNA-transfected or control siRNA-transfected cells for all doses tested. The lack of functional effect of MMP-9 silencing in Mock-cells could be explained by the very low constitutive levels of MMP-9 in these cells (see Figure 8E). Thus, modulation of these levels is therefore unlikely to produce a detectable functional effect. In contrast, silencing MMP-9 in MMP-9-cells significantly decreased cell viability with respect to control siRNA-transfected cells, thus reverting the protective effect of MMP-9 on these cells (Figure 8F). These results clearly indicated that MMP-9 contributed to CLL cell resistance to ATO.

### MMP-9 induces drug resistance by modulating the balance of anti- and pro-apoptotic proteins from the Bcl-2 family

**Figure 9:**
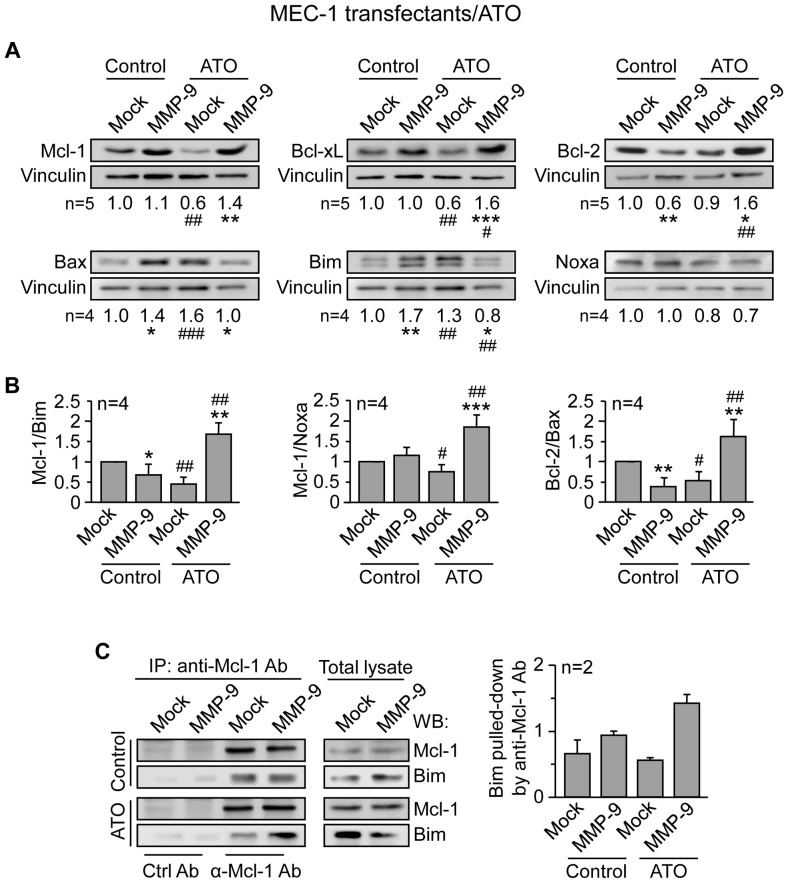
MMP-9 expression in MEC-1 cells prevents downregulation of anti-apoptotic Bcl-2 family proteins in response to ATO. (A) 5×10^6^ Mock- or MMP-9-MEC-1 cells were treated or not with 5 µM ATO. After 24 h cells were lysed and the expression of the indicated proteins was analyzed by Western blotting, using vinculin as an internal control. A representative experiment is shown for each case and numbers indicate the average values from all experiments performed, after normalizing Mock control values to 1. (B) The indicated ratios of anti-apoptotic/pro-apoptotic proteins are shown. (C) 3×10^7^ Mock- or MMP-9-cells were treated or not with 5 µM ATO for 24 h. Cells were lysed and lysates immunoprecipitated with anti-Mcl-1 or control Abs and analyzed by Western blotting. Values indicate the amount of Bim found in the Mcl-1 immunoprecipitates in both types of MEC-1 transfectants. * or ^#^P≤0.05; ** or ^##^P≤0.01; *** or ^###^P≤0.001. Symbols are: *, Mock- vs MMP-9-cells; ^#^, Mock- or MMP-9-cells treated with ATO compared to their respective untreated counterparts.

To determine the molecular bases responsible for the observed drug-protecting effect of MMP-9, we analyzed whether the expression of prototype Bcl-2 family members was modulated by MMP-9. In initial experiments, Mock- and MMP-9-cells were treated with 5 µM ATO or vehicle and, after 24 h, lysed and analyzed by Western blotting. Figure 9A shows that upon ATO exposure, expression of the anti-apoptotic proteins Mcl-1 and Bcl-xL decreased in Mock-cells compared to their untreated counterpart, while Bcl-2 remained unchanged. In MMP-9-cells, however, all three proteins remained similar (Mcl-1) or were elevated (Bcl-xL, Bcl-2) with respect to untreated MMP-9-cells. Moreover, Mcl-1, Bcl-xL and Bcl-2 in ATO-treated MMP-9-cells were significantly upregulated compared to ATO-treated Mock-cells (Figure 9A). Similar analysis of selected pro-apoptotic proteins showed that Bax and Bim significantly increased in Mock-cells treated with ATO, compared to untreated cells, while Noxa expression was not significantly altered (Figure 9A). In contrast, the expression of Bax and Bim in MMP-9-cells treated with ATO was lower than in untreated MMP-9-cells. Moreover, Bax and Bim in ATO-treated MMP-9-cells were significantly lower than in ATO-treated Mock-cells (Figure 9A).

Since regulation of apoptosis/survival involves the balance of anti- and pro-apoptotic Bcl-2 family members, rather than individual levels [27], we also determined the ratios of these proteins in Mock and MMP-9-cells, before and after exposure to ATO. Figure 9B shows that, upon ATO treatment, the balance Mcl-1/Bim, Mcl-1/Noxa and Bcl-2/Bax, were all significantly increased in MMP-9-cells, compared to Mock-cells. These ratios were also significantly higher compared to untreated MMP-9-cells. However, these ratios were diminished in ATO-treated Mock-cells, compared to untreated Mock-cells (Figure 9B).

Mcl-1 is a critical molecule in CLL cell survival and exerts its function by sequestering BH3-only pro-apoptotic proteins such as Bim [28]. We therefore studied whether Mcl-1 and Bim were complexed in MMP-9 transfectants upon ATO exposure. Lysates of Mock or MMP-9-cells that had been treated or not with 5 µM ATO for 24 h, were immunoprecipitated with anti-Mcl-1 Abs and analyzed by Western blotting. Figure 9C shows that in untreated cells, similar amounts of Bim were pooled down by the anti-Mcl-1 Ab in both, Mock and MMP-9 transfectants. In contrast, upon ATO treatment, very little Bim co-immunoprecipitated with Mcl-1 in Mock-cells, while Bim remained bound to Mcl-1 and even increased in MMP-9 cells (Figure 9C).

**Figure 10:**
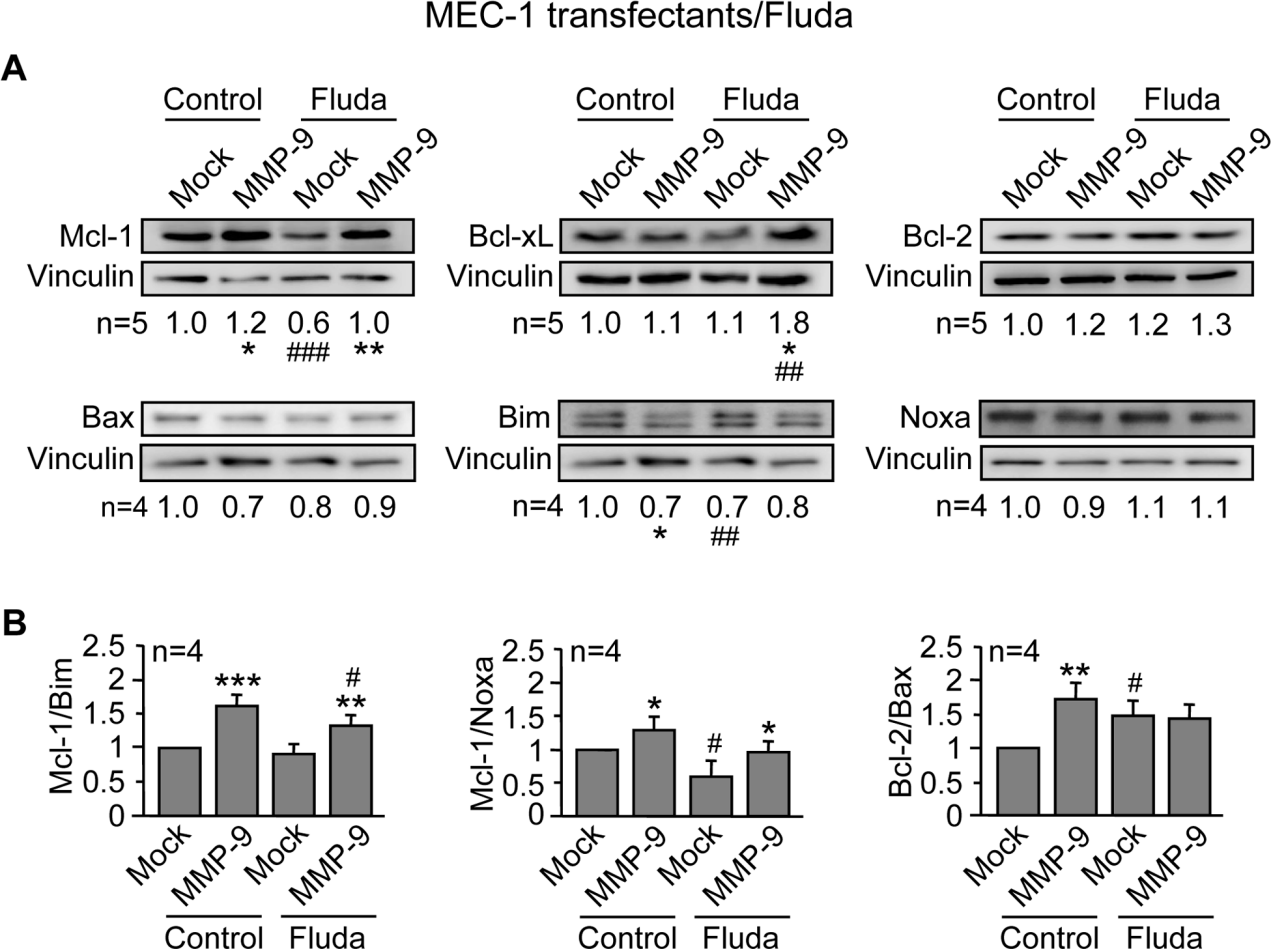
MMP-9 expression in MEC-1 cells prevents downregulation of anti-apoptotic Bcl-2 family proteins in response to fludarabine. (A,B) 5×10^6^ Mock- or MMP-9-cells were treated or not with 5 µM fludarabine (Fluda). After 48 h cells were lysed and the indicated proteins (A) and ratios (B) analyzed as in Figure 9. * or ^#^P≤0.05; ** or ^##^P≤0.01; *** or ^###^P≤0.001. Symbols are: *, Mock- vs MMP-9 cells; ^#^, Mock- or MMP-9-cells treated with Fluda compared to their respective untreated counterparts.

Similar results were obtained for Mock- and MMP-9-cells treated with 5 µM fludarabine. As shown in Figure 10A, Mcl-1 and Bcl-xL were also significantly higher in MMP-9-cells compared to Mock-cells. Bcl-2 did not seem to play a role in this case, as its expression remained similar for both cell types, either in the absence or presence of fludarabine. As observed in the case of ATO, the ratios Mcl-1/Bim and Mcl-1/Noxa upon fludarabine treatment were significantly higher in MMP-9-cells than in Mock-cells, while the balance Bcl-2/Bax did not seem to play a role (Figure 10B).

### MMP-9 regulates the balance of Bcl-2 family proteins in primary CLL cells treated with ATO

**Figure 11:**
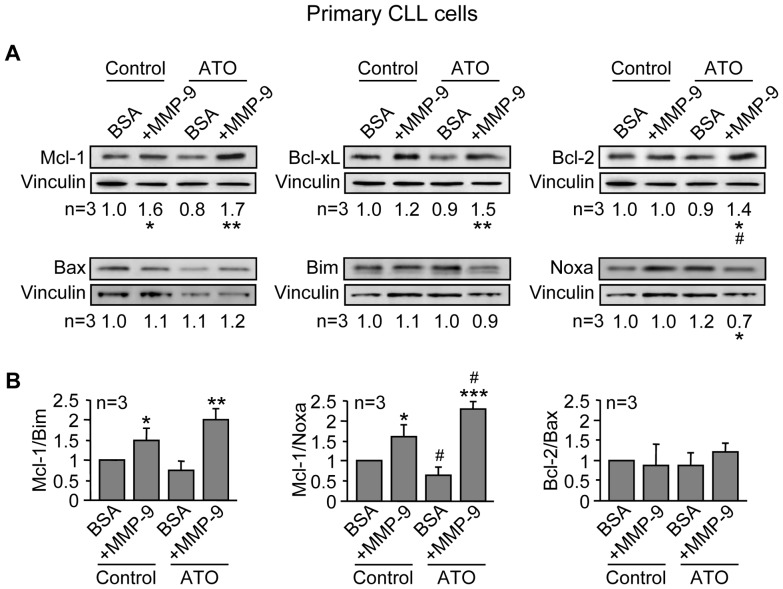
Culturing CLL cells on MMP-9 modulates Bcl-2 family proteins in response to ATO, preventing downregulation of Mcl-1, Bcl-xL and Bcl-2. (A,B) 10×10^6^ primary CLL cells in RPMI/0.1% FBS were incubated on BSA- or 150 nM MMP-9-coated wells for 1 h prior to adding 3 µM ATO or vehicle. After 24 h, cell were lysed and the indicated proteins (A) and ratios (B) analyzed by Western blotting as explained. * or ^#^P≤0.05; ** or ^##^P≤0.01; *** or ^###^P≤0.001. Symbols are: *, CLL cells on BSA vs CLL cells on MMP-9; ^#^, ATO-treated cells compared to their respective untreated controls.

We next determined whether MMP-9 also modulated Bcl-2 family members in primary CLL cells. Cells from 3 different patients were cultured on BSA or MMP-9 for 1 h prior to adding 3 µM ATO. After 24 h, cells were lysed and lysates analyzed by Western blotting. Figure 11A shows that Mcl-1 was significantly upregulated, both in the absence or presence of ATO, in cells cultured on MMP-9 compared to cells cultured on BSA. Bcl-xL and Bcl-2 were also significantly increased on MMP-9-cultured CLL cells upon ATO treatment, compared to BSA-cultured cells. The ratios Mcl-1/Bim and Mcl-1/Noxa were also upregulated in MMP-9-cultured cells, both in the absence or presence of ATO, while the Bcl-2/Bax ratio was not modulated under these conditions (Figure 11B). Altogether these results indicated that MMP-9 induced drug resistance in CLL cells by regulating the expression and function of crucial anti-apoptotic and pro-apoptotic proteins of the Bcl-2 family.

## Discussion

We have studied whether MMP-9 plays a role in CLL cell response to cytotoxic drugs, such as arsenic trioxide and fludarabine. We report for the first time the following findings: 1) upon an apoptotic stimulus, MMP-9 is transcriptionally upregulated and localizes to the surface of early apoptotic cells; 2) MMP-9 by itself or present in stroma induces CLL cell drug resistance; 3) MEC-1 cells stably transfected with MMP-9 show increased survival upon drug treatment; 4) the MMP-9 anti-apoptotic effect involves modulation of anti- and pro-apoptotic proteins from the Bcl-2 family.

Upregulation and membrane localization of MMP-9 was an early response to drug exposure that preceded detection of apoptosis and was necessarily associated to this process. Evidence for this comes from the fact that preventing cell death with the Z-VAD-FMK caspase inhibitor blocked MMP-9 mRNA induction and its cell surface localization upon ATO treatment. MMP-9 membrane association was a specific event and involved interaction with α4β1 integrin and CD44, two reported MMP-9 receptors in CLL [16]. Additionally, the cell population with increased surface MMP-9 was coincident with that of early apoptotic cells, suggesting that MMP-9 remained mostly surface-bound to these cells upon secretion and modulated the apoptotic response. As MMP-9, as other MMPs, may play dual roles in apoptosis [18], [19], one interpretation of our results could be that upregulation of MMP-9 facilitates the apoptotic process by targeting appropriate substrates or pathways. An alternative explanation is that MMP-9 antagonizes the apoptotic cell response to cytotoxic drugs, thus representing a survival compensatory mechanism. In support of this explanation, several previous studies have reported compensatory or survival response for MMP-9 in various cell systems. For example, in melanoma and breast carcinoma cells, apoptosis induced by TNF receptor ligands was clearly enhanced by inhibiting MMP-9 (or MMP-2) [29]. Similarly, blocking MMP-9 function sensitized colon adenocarcinoma cells to phorbol-esters [30] and glioma cells to Fas-induced apoptosis [31]. In another report using a xenograft model in mice, treatment of carcinoma cells with the chemotherapeutic agent paclitaxel increased MMP-9 expression and tumor cell metastasis, and this was also blocked with an MMP-9 inhibitor [32].

Using two different approaches we have obtained strong evidence to support an anti-apoptotic role for MMP-9 in the CLL cell response to ATO and fludarabine. First, the higher viability observed on cells cultured on MMP-9 during drug treatment compared to cells cultured on BSA. Notably, this anti-apoptotic role of MMP-9 was also observed in co-cultures of CLL and stromal cells, where blocking MMP-9 with antibodies completely reverted the stroma-induced drug resistance. We previously reported a role for MMP-9 in the protective effect of stroma against CLL cell spontaneous apoptosis in culture [17]. Other investigators have shown the involvement of several proteins (integrins, chemokines, Bcl-2 family proteins) in the resistance to certain therapeutic agents induced by stroma [33]–[35]. We now show for the first time that stromal cells induce CLL cell resistance to ATO and that MMP-9 has a prominent role in this resistance.

Further evidence for a survival role for MMP-9 in response to cytotoxic drugs comes from the fact that MEC-1-MMP-9 transfectants, representing an unambiguous system to identify MMP-9 functions, consistently showed higher viability in the presence of ATO or fludarabine than their corresponding MEC-1-Mock controls. Indeed, this effect was mediated by MMP-9 as silencing this protein reverted the survival advantage of the MMP-9 transfectants. Since our results show that MMP-9 is present in these transfectants as a cell-associated form as well as in the conditioned medium, it is possible that both fractions contribute to the increased survival of these cells. It is not known if the MMP-9 survival effect involves the same or different mechanisms as MMP-9 upregulation upon apoptotic stimuli, but the results of our study strongly support a compensatory survival role for MMP-9 in CLL.

We have addressed the molecular bases accounting for this drug-resistance effect of MMP-9 and have focused on molecules from the Bcl-2 family, well-known regulators of apoptosis [27]. The apoptotic action of ATO has been shown to involve downregulation of the anti-apoptotic protein Mcl-1 in several cell systems, including myeloma [36] and myeloid leukemia cells [37] and, in many cases, upregulation of the pro-apoptotic proteins Bax and/or Bim [36], [38], [39]. Indeed the balance Mcl-1/Bim was shown to be determinant in myeloma cell response to ATO [36] and in the resistance of acute and chronic leukemic cells to fludarabine [40]. Our present results clearly show that MMP-9, both in MEC-1 transfectants and in primary CLL cells, not only prevented downregulation of anti-apoptotic proteins (Mcl-1, Bcl-xL, Bcl-2) in response to ATO but also upregulated their levels with respect to basal expression. As this was accompanied by downregulation (or no alteration) of pro-apoptotic proteins (Bim, Bax), the anti-apoptotic/pro-apoptotic balance was clearly elevated in the presence of MMP-9, likely contributing to the MMP-9 survival effect. We previously reported that prevention of CLL cell spontaneous apoptosis by MMP-9 involved upregulation of Mcl-1 but not Bcl-xL or Bcl-2 [17]. We now show that, in the presence of ATO, MMP-9 seems to affect several of these proteins, perhaps to amplify the compensatory survival effect. In this regard, our results also indicate that Mcl-1, a crucial anti-apoptotic protein in CLL [28] was not dissociated from Bim upon ATO exposure on MMP-9-transfected cells, thus preventing Bim from causing mitochondrial damage and apoptosis. Our results further show that Mcl-1 and Bcl-xL were also upregulated by MMP-9 in response to fludarabine. This is in agreement with our previous report showing the involvement of these proteins in the fibronectin/α4β1 integrin-induced CLL cell resistance to fludarabine [41]. As in the case of ATO, the anti-apoptotic/pro-apoptotic protein ratio was elevated, suggesting that the protective effect of MMP-9 against apoptosis may be a general CLL cell response to cytotoxic drugs. In conclusion, our study is the first to establish that MMP-9 induces drug-resistance in CLL by modulating the balance of Bcl-2 family members. Targeting MMP-9 in combination with therapeutic agents may thus improve the CLL response to treatment.
